# Expression Profiles of Long Noncoding RNAs and Messenger RNAs in a Rat Model of Spinal Cord Injury

**DOI:** 10.1155/2023/6033020

**Published:** 2023-01-19

**Authors:** Jian Cao, Tao Tang, Jianye Tan, Qi Chen, Jinghong Yuan, Tao Li, Xigao Cheng

**Affiliations:** ^1^Department of Orthopedics, The Second Affiliated Hospital of Nanchang University, 330006, 1 Minde Road, East Laker District, Nanchang, China; ^2^Institute of Orthopedics of Jiangxi Province, Jiangxi 330006, 1 Minde Road, East Laker District, Nanchang, China; ^3^Institute of Minimally Invasive Orthopedics, Nanchang University, Jiangxi 330006, 1 Minde Road, East Laker District, Nanchang, China; ^4^Jiangxi Key Laboratory of Intervertebral Disc Disease, Nanchang University, Jiangxi 330006, 1 Minde Road, East Laker District, Nanchang, China

## Abstract

Spinal cord injury (SCI) is a serious disorder of the central nervous system with a high disability rate. Long noncoding RNAs (lncRNAs) are reported to mediate many biological processes. The aim of this study was to explore lncRNA and mRNA expression profiles and functional networks after SCI. Differentially expressed genes between SCI model rats and sham controls were identified by microarray assays and analyzed by functional enrichment. Key lncRNAs were identified using a support vector machine- (SVM-) recursive feature elimination (RFE) algorithm. A trans and cis regulation model was used to analyze the regulatory relationships between lncRNAs and their targets. An lncRNA-related ceRNA network was established. We identified 5465 differentially expressed lncRNAs (DE lncRNAs) and 8366 differentially expressed mRNAs (DE mRNAs) in the SCI group compared with the sham group (fold change > 2.0, *p* < 0.05). Four genes were confirmed by qRT-PCR which were consistent with the microarray data. GSEA analysis showed that most marked changes occurred in pathways related to immune inflammation and nerve cell function, including cytokine-cytokine receptor interaction, neuroactive ligand-receptor interaction, and GABAergic synapse. Enrichment analysis identified 30 signaling pathways, including those associated with immune inflammation response. A total of 40 key lncRNAs were identified using the SVM-RFE algorithm. A key lncRNA-mRNAs coexpression network was generated for 230 951 lncRNA-mRNA pairs with half showing positive correlations. Several key DE lncRNAs were predicted to have “cis”- or “trans”-regulated target genes. The transcription factors, Sp1, JUN, and SOX10, may regulate the interaction between XR_001837123.1 and ETS 1. In addition, five pairs of ceRNA regulatory sequences were constructed. Many mRNAs and lncRNAs were found to be dysregulated after SCI. Bioinformatic analysis showed that DE lncRNAs may play crucial roles in SCI. It is anticipated that these findings will provide new insights into the underlying mechanisms and potential therapeutic targets for SCI.

## 1. Introduction

Spinal cord injury (SCI) is a serious form of central nervous system injury, frequently caused by falls, car accidents, or sports injuries. SCI can be devastating when it leads to irreversible impairment of the motor, sensory, and autonomic functions of the spinal cord and can seriously affect the patient's mental health. In the world, 250 000 to 500 000 new cases of SCI occur every year, according to the World Health Organization (WHO) [1]. If the injury is located in the upper cervical region, the patient may have quadriplegia. In contrast, injuries at the chest or waist level can lead to paralysis of the patient's lower limbs, known as paraplegia. SCI is associated with a variety of complex processes pathogenic mechanisms, including cellular edema, neuronal death, oxidative stress, and inflammation, that complicate treatment and management [2].

Many studies have been conducted on the molecular mechanisms of glial cell activation and polarization, neuronal death, and glial scar formation caused by SCI. Recent studies have demonstrated the involvement of many genetic and signaling pathways in SCI, some of which may become key therapeutic targets. These include SARM1 [3], CCL3 [4], the PI3K/AKT/Foxo1 pathway [5], Nrf2/HO-1 signaling [6], and SIRT-1/NF-*κ*B signaling [7]. However, despite the identification of various molecular and cellular mechanisms involved, much of SCI remains a mystery.

A comprehensive understanding of transcriptional regulation is essential for understanding most biological processes. The role of lncRNAs in various cellular processes cannot be overstated; these include transcription, posttranscription, translation, and epigenetic regulation [8]. lncRNAs are expressed in the nervous system where they act as sponges to capture miRNA and interact with RNA-binding proteins [8]. There is significant evidence that the lncRNAs KCNQ1OT1 [9] and Malat1 [10] function as competing endogenous RNAs and influence the recovery process after traumatic brain injury. It is thus reasonable to speculate that after SCI, there will be significant changes in the expression levels of lncRNA in the cells and tissues, and some of these specific changes may provide new directions for treating SCI.

In this study, we used the Agilent Rat ceRNA Microarray to screen differentially expressed lncRNAs and mRNAs (DE lncRNAs and mRNAs, respectively) in injured spinal cord tissue in rats. Coexpression of genes and functional and pathway enrichment analyses were used to annotate the functions of key lncRNAs. A cis/trans regulation analysis was also conducted to analyze the relationships between lncRNAs and mRNAs. As well, a ceRNA regulatory network related to lncRNAs was established.

## 2. Materials and Methods

### 2.1. Rat SCI Model

The Nanchang University Animal Center provided us with male Sprague Dawley (SD) rats weighing 200 g to 250 g. The Ethics Committee of the Second Affiliated Hospital of Nanchang University approved the study. Six rats were maintained for a minimum of one week until the model surgery was performed. Three rat SCI models were established by the modified Allen method. Inhalation of isoflurane was used to anesthetize rats and place them on an operating table in prone position. The head and limbs were held in place, and the fur along the back was removed for skin preparation. After strict disinfection, a median incision of approximately 3 cm in length was made at the T10 level. The skin, subcutaneous tissue, and myofascia were carefully separated and dissected, exposing and fenestrating the T10 lamina. The spinal cord was carefully exposed without damaging the dura mater. A heavy hammer weighing 10 g with a diameter of 2.5 mm was allowed to fall freely from a height of 8 cm, fully impacting the dural sac; the hammer was then retracted immediately after SCI. After the blow, the rats show a tail-wagging reflex with twitching and stretching of both hind limbs and the body; this is a stress reflex and indicates the success of the modeling. The sham operation group received the same procedure without injury to the spinal cord.

### 2.2. Microarray and Data Analyses

Six male Sprague Dawley (SD) rats were analyzed using the Agilent Rat ceRNA Microarray 2019 (8 ^∗^ 60K, design ID: 086243) chip. Three days after SCI, we extracted spinal cord RNA using TRIzol reagent (Thermo Fisher Scientific, Waltham, MA, USA). The RNA purity and concentration were determined using the Nanodrop ND-2000 (Thermo Fisher). The total RNA was reverse-transcribed to cDNA, and cRNA was synthesized and labeled with cyanine-3-CTP. The microarray chipsets were hybridized with the labeled cRNAs. A single array of hybridized mRNA plus lncRNA was scanned using an Agilent Scanner G2505C (Agilent Technologies, CA, USA).

Analyzing the array data was performed with GeneSpring software v13.0 (Agilent Technologies). The probes with more than 75% samples of one group flagged “detected” were selected for further data analysis. A quantile algorithm was used to normalize the raw data. The threshold values for DE mRNA and DE lncRNA between the two groups were ≥2 or ≤ −2 fold change (FC), *p* < 0.05. A hierarchical clustering technique was used to evaluate DE gene clustering patterns on the same platform. China's OEbiotech (Shanghai, China) conducted the data analysis. The array of microarray data has been deposited in Gene Expression Omnibus (GEO, https://www.ncbi.nlm.nih.gov/geo/), with accession number GSE218088.

### 2.3. Screening Key Differentially Expressed lncRNAs

To extract DE lncRNAs with high prediction accuracy, the support vector machine- (SVM-) recursive feature elimination (RFE) algorithm was used for identifying key DE lncRNAs. Specifically, SVM-RFE recursively removes ground influence factors allowing for better genetic screening. This method was implemented using the cfe function in the “caret” package (6.0-93), and the fitted prediction function was cross-validated.

### 2.4. lncRNA Coexpression Analysis and Gene Functional Annotation

An analysis of volcano plots was used to identify lncRNAs and mRNAs that had statistically significant expression differences. Hierarchical clustering was used to analyze diacritical lncRNA and mRNA expression patterns. GO (http://geneontology.org/) and KEGG (http://www.genome.jp/kegg/) pathway enrichment analyses were also performed on DE mRNAs to gain a deeper understanding of their roles. The DE lncRNAs were classified to explore their potential functions.

Coexpression networks were constructed from DE mRNAs and lncRNAs. The Pearson correlation coefficient between two genes had to be greater than 0.99 to be considered significant. Cytoscape (v3.7.2) was used to construct coexpression networks of mRNAs and lncRNAs that were significantly correlated.

### 2.5. GSEA

Gene Set Enrichment Analysis (GSEA, v2.2.2) is a powerful and flexible analytic method that can determine whether a previously defined group of genes (rather than a single gene) show consistent significant differences between two biological states. The GSEA does not only consider genes with significant DE but all genes. In GSEA, significance is assessed by permuting the class labels, which preserves gene correlations and thus provides a more accurate null model. We performed GSEA to investigate whether GO terms or pathways associated with specific genes differed between the two groups.

### 2.6. Target Prediction

Both “cis” and “trans” regulatory mechanisms are used for regulating lncRNAs. The presence of cis regulation was predicted for coexpressed mRNA-lncRNA pairs with Pearson's correlation coefficients greater than 0.99, while the FEELnc software (v 0.2.1) was used to identify possible mRNA-binding sites in the 100 kb window upstream or downstream of lncRNA [11]. Prediction of trans regulation was performed using RIsearch-2.0 (https://rth.dk/resources/risearch/) [12]. Using its screening conditions, this predicted that more than 10 bases are required for direct nucleic acid interaction between coexpressed lncRNAs and mRNAs; in addition, the binding free energy of the base was not greater than -100 [13]. The screened interacting lncRNAs and mRNAs may be directly regulated. To identify potential regulatory targets, the JASPAP (http://jaspar.genereg.net/) and GTRD (v 16.07) databases were used to identify interactions between lncRNAs and their transcription factors (TFs) [14, 15]. Network software package in R (v 3.2.0) was used to construct the TF-lncRNA-mRNA network.

### 2.7. Targeted DE lncRNAs Associated ceRNA Network Construction

Predictions of lncRNA/mRNA and miRNA binding to targets were performed using miRBase22 (https://www.mirbase.org/) with a binding free energy of less than -20. DE lncRNAs and DE mRNAs were defined as those showing greater than 0.99 relative |PCC| expression values. A ceRNA network was then constructed using lncRNA-miRNA and miRNA-mRNA regulatory pairs. With the exception of overlapping miRNA binding, Cytoscape (v 3.7.2) identified mRNAs with the same expression patterns as lncRNAs to construct an lncRNA-miRNA-mRNA ceRNA network.

### 2.8. qPCR

Spinal cord tissues were assayed for lncRNA expression using quantitative real-time PCR (qRT-PCR). We chose two lncRNAs (NONRATT019701.2 and XR_001838273.1) with potential regulatory relationships with the Cdca3 and F10 genes, respectively. TRIzol (Takara, Japan) was used to extract total RNA from spinal cord samples. Spinal cord RNA was reverse-transcribed to cDNA using PrimeScript™ RT Master Mix (Takara, Japan). SYBR Premix Ex Taq II kits (Takara, Japan) were used for the RT-qPCR analysis as carried out in accordance with the manufacturer's instructions. The data were analyzed by the 2-△△CT method. The lncRNAs and mRNAs primers are listed in Supplementary Table 1.

## 3. Results

### 3.1. Identification of DE lncRNAs and mRNAs in SCI

**Figure 1:**
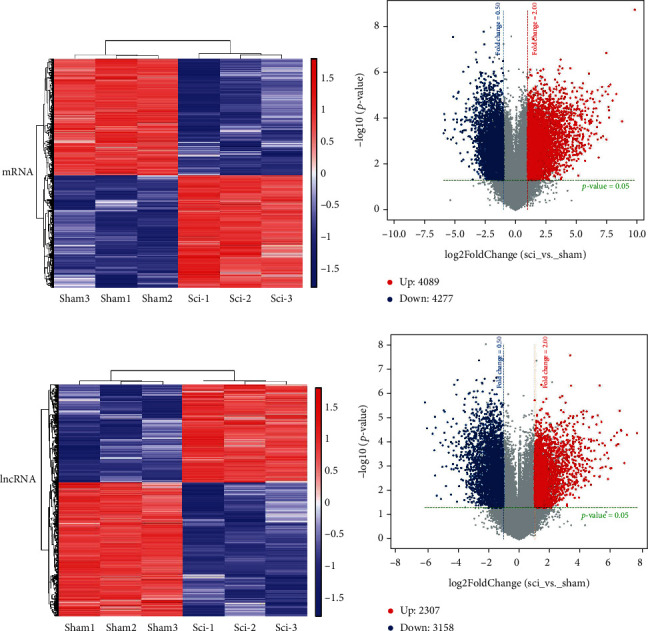
DE lncRNAs and DE mRNAs in the SCI and sham groups. DE mRNAs and lncRNAs in the sham and SCI groups are displayed as heatmaps (a, c). Colors indicate different clusters in the data. Volcano plot of DE mRNAs and lncRNAs (b, d). Dots in red indicate genes that have been significantly upregulated, while dots in blue indicate genes that have been significantly downregulated. Volcano plot of 5465 DE lncRNAs and 8366 DE mRNAs in SCI with statistical significance (∣FC | >2.0, *p* < 0.05).

Using microarray analysis, we determined the mRNA and lncRNA expression profiles in SCI rats. We show the distribution of the two sets of data before and after normalization through box plots in order to understand the distribution of the data (Supplement Figure 1). Specifically, an FC of >2 and a *p* < 0.05 were considered significant threshold values for defining upregulation or downregulation of genes, and cluster maps and volcano plots were used to compare the mRNA and lncRNA expression profiles between the SCI and sham groups (Figure 1).

In total, 5465 DE lncRNAs were identified. Of these, 2307 were found to be upregulated in the SCI group while 3158 DE lncRNAs were downregulated (Table 1). The top five significantly upregulated DE lncRNAs, with log2FC values ranging from 7.6 to 6.6, were NONRATT006251.2, XR_590951.1, NONRATT021749.2, NONRATT000346.2, and NONRATT006253.2. The top five most significantly downregulated DE lncRNAs, with log2FC values ranging from -6.1 to -5.4, were XR_001838225.1, NONRATT006738.2, NONRATT014374.2, NONRATT024533.2, and XR_001836692.1 (Table 1).

We then identified 8366 DE mRNAs by comparing the total mRNA changes between the two groups. Of these, 4089 were upregulated, and 4277 were downregulated (Figure 1). The five most significantly upregulated DE mRNAs in the SCI group were XM_006236630.3, ENSRNOT00000009392, XM_017592759.1, ENSRNOT00000077186, and ENSRNOT00000077158, all of which showed log2FC values between 9.7 and 7.8. The five most significantly downregulated DE mRNAs in the SCI group were ENSRNOT00000089734, XM_006232396.2, ENSRNOT00000034394, ENSRNOT00000086508, and ENSRNOT000000881, which showed log2FC values between -5.9 and -5.6 (Table 1).

### 3.2. Functional Enrichment Analysis of DE mRNAs

**Figure 2:**
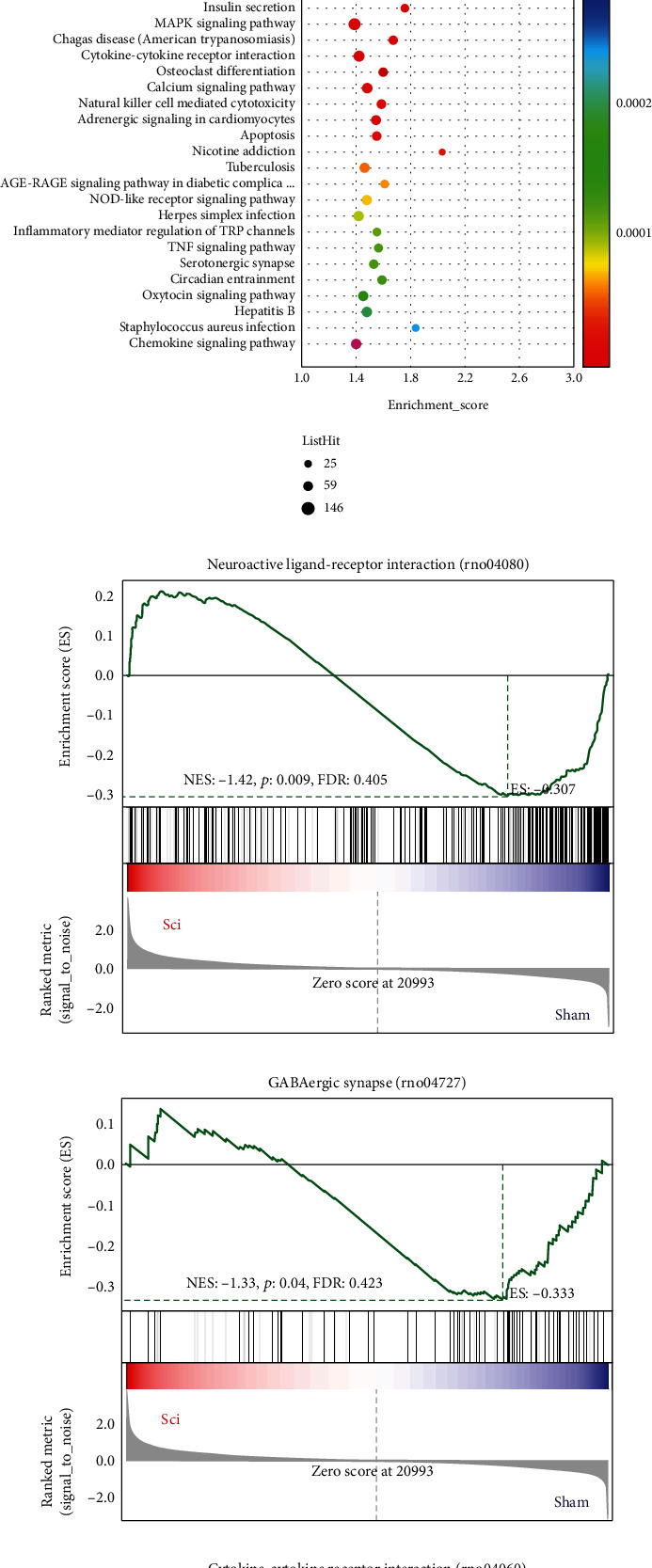
Functional enrichment analysis of the DE mRNAs. Top 10 enriched GO terms in the molecular function, biological process, and cellular component annotations for DE mRNAs (a). Top 30 enriched KEGG pathways for DE mRNAs (b). GSEA results of genes related to nerve cell function and immune inflammation (c–f).

The enrichment of DE mRNAs in GO and KEGG pathways was then investigated to predict their functions and associated pathways. The GO analysis of DE mRNAs identified changes in the cellular component (CC), biological process (BP), and molecular function (MF) GO categories. The 30 most significantly enriched GO terms are shown in Figure 2(a). It can be seen that the DE mRNAs were mainly enriched in the BPs of potassium ion transmembrane transport, inflammatory response, and regulation of ion transmembrane transport. The most important MFs were related to protein binding, cell adhesion molecule binding, and identical protein binding. Of the CCs, the significantly enriched GO terms included integral component of plasma membrane, cell surface, and synapse.

KEGG analysis identified a number of significantly enriched pathways associated with SCI, as shown in Figure 2(b); these included neuroactive ligand-receptor interaction, cholinergic synapse, lysosome, NF-*κ*B signaling pathway, and glutamatergic synapse. Supplementary Figure 2 provides greater detail on these enriched terms and pathways.

In order to further explore the enrichment and analysis of DEGs in the SCI and sham groups, we analyzed their potential molecular functions through GSEA and found that neuroactive ligand-receptor interaction (rno04080)- and GABAergic synapse (rno04727)-related genes were significantly downregulated in the SCI group, while cytokine-cytokine receptor interaction (rno04060)- and TNF signaling pathway (rno04668)-related genes were significantly upregulated (Figure 2(c)). These results indicate that SCI is associated with a shift in the immune response toward a proinflammatory state, together with a sharp decline in pathways related to nerve cell function.

### 3.3. Identification of Key lncRNAs

**Figure 3:**
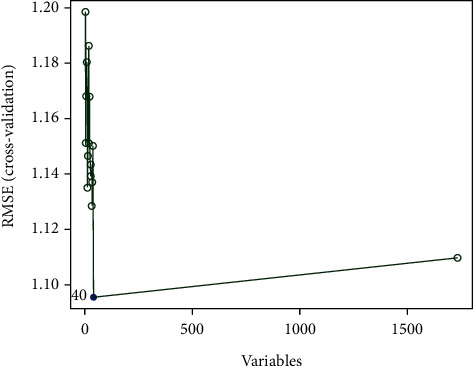
SVM-RFE algorithm results. A distribution of the relationship between RMSE and the number of lncRNAs. The horizontal axis shows the number of lncRNAs, and the vertical axis shows the RMSE corresponding to the number of lncRNAs.

The SVM-RFE algorithm was utilized for dimensionality reduction screening of the DE lncRNAs. As described in Materials and Methods, lncRNAs with the lowest RMSE (standard error) values in the model were selected as key lncRNAs. This resulted in the identification of 40 key lncRNAs (Figure 3 and Supplementary Table 2).

### 3.4. Construction of lncRNA-mRNA Coexpression Network

**Figure 4:**
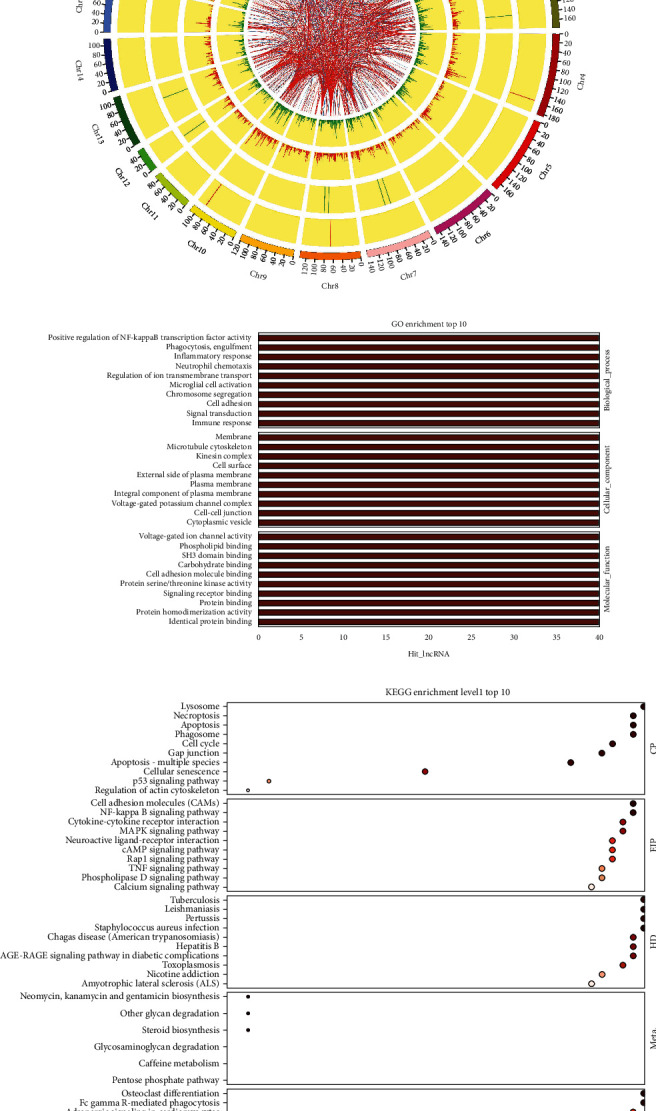
lncRNA-mRNA coexpression network in SCI of rat model. Coexpression circos map: the outermost circle is the autosome distribution diagram of this species; the second and third circles are the distribution of DE genes on chromosomes. The red line indicates that genes are upregulated, and the green line indicates that genes are downregulated. The higher the column, the more DE genes there are. In the fourth and fifth circles, DE lncRNAs were shown in the same form as genes. The corresponding relationship between top 500 coexpressed key lncRNAs and genes is indicated by the internal line (a). The GO enrichment results of key lncRNA coexpressed DE mRNAs correspond to the top 10 terms (b). The KEGG enrichment results of total key lncRNA coexpressed DE mRNAs correspond to the top 10 terms (c). The *x* axis represents the enrichment score. More differential genes are present in items with larger bubbles. Bubble color changes from gray to red, with a gradually decreasing enrichment *p* value, indicating increasing significance.

Based on the correlation analysis between DE mRNAs and DE lncRNAs, a key lncRNA-mRNA coexpression network was constructed to explore gene interactions in SCI. Significant mRNA-lncRNA pairs (of no more than 6000 nucleotides in length) with Pearson's correlations of no less than 0.99 and *p* values > 0.05 were selected. This identified 230 951 key lncRNA-mRNA pairs with coefficient scores > 0.99. Most of these (115 244/230 951) were positively associated (Figure 4(a)). Circos diagrams were generated with drawing software to display the comparison between the groups in a more intuitive manner (Figure 4(a)).

Each key lncRNA coexpressed with an mRNA was analyzed for functional and KEGG pathway enrichment analyses. It is possible that lncRNAs function in a similar manner to the GO and KEGG enrichment analyses of genes. The top three GO results were positive regulation of NF-*κ*B transcription factor activity, phagocytosis engulfment, and inflammatory response in the BP category; membrane, microtubule cytoskeleton, and kinesin complex in CC; and voltage-gated ion channel activity, phospholipid binding, and SH3 domain binding in MF (Figure 4(b)).

We then investigated KEGG pathway enrichment analysis in relation to the first category (cellular processes, environmental information processing, genetic information processing, human diseases, metabolites, and biological system). Each class of dysregulated mRNA was found to be associated with the top 10 pathways, as shown in Figure 4(c). The top pathways for cellular processes included apoptosis, lysosomes, and necroptosis. In terms of environmental information processing, the top pathways were cell adhesion molecules, NF-*κ*B signaling pathway, and cytokine-cytokine receptor interaction. These results indicate that the candidate genes were associated with the top pathways, especially those related to cell death and immune inflammation.

### 3.5. “Cis” Analysis of the DE Key lncRNA and Adjacent Coexpression DE mRNA in SCI

**Figure 5:**
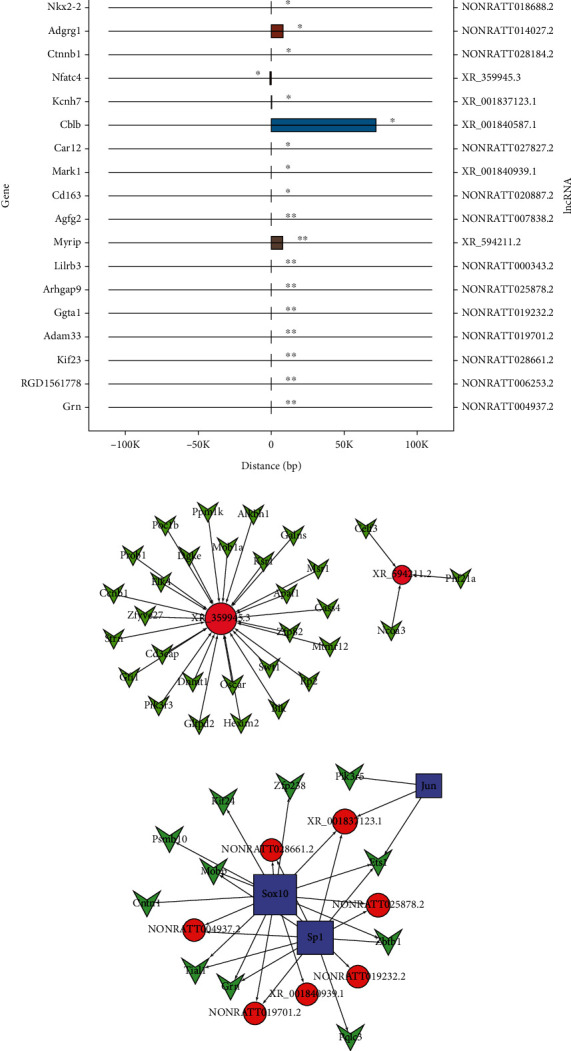
“Trans,” “cis,” and “TF” mechanism of the aberrant lncRNAs. On the graph, the data points on the left and right of the *y* axis correspond to mRNA and lncRNA, respectively; ^∗^*p* < 0.05 and ^∗∗^*p* < 0.01. The *x* axis represents the distance between mRNA and lncRNA, the negative value represents upstream, the positive value represents downstream, and the same color bar graph represents same lncRNAs (a). lncRNA is represented by the red node and mRNA by the green node, and the number is represented by the size of the node (b). A node of red color represents lncRNA, a node of green color represents mRNA, and a node of blue color represents TF. Only nodes with degree > 2 are shown (c).

lncRNAs may cis regulate coding genes that are located close to the lncRNAs and with which they are coexpressed. Therefore, we screened the adjacent genes of the dysregulated key lncRNAs with stringent selection criteria (intergenic distance < 100 kb). After comparison between the SCI and sham groups, 20 top cis-regulated lncRNAs and mRNAs were identified in the SCI group, as shown in Figure 5(a). Among these “cis” genes, Adgrg1 is associated with oligodendrocyte precursor cell proliferation and the number of myelinated axons [16], and NFATc4 is associated with neuronal apoptosis [17].

### 3.6. Trans Regulation of Key DE lncRNAs in SCI and Construction of a Key lncRNA-TF-Target Gene Network

A diagram of the top 500 key lncRNA-mRNA pairs was compiled using the network software package (Figure 5(b)). lncRNA XR_359945.3 was predicted to transregulate 27 mRNAs, including Msr1, Ccnb1, and Apaf1, while lncRNA XR_594211.2 was predicted to transregulate three mRNAs, namely, Ncoa3, Phf21a, and Celf3.

Using the gene-TF pairs identified by GTRD database and the top 500 key lncRNA-mRNA coexpression pairs, we used network software to create a ternary regulatory network of lncRNAs, TFs, and mRNAs and draw a ternary regulatory network diagram. This analysis used the top 20 cis-related key lncRNAs and their coexpression of mRNAs.  Figure 5(c) shows the core TF–lncRNA–target gene relationship map showing the differences between the SCI and sham-operation groups. This core map contains 7 lncRNAs, 10 target genes, and 3 core TFs. Five lncRNAs and six mRNAs were found to be modulated by the TF Sp1, whereas four lncRNAs and eight mRNAs were modulated by SOX10. Specifically, it was found that Sp1, JUN, and SOX10 can simultaneously regulate lncRNA XR_001837123.1 and *Ets1*. This map allows a more comprehensive understanding of the relationships between lncRNAs, target genes, and transcription factors, as well as additional information for future studies.

### 3.7. Construction of a ceRNA Regulatory Network

**Figure 6:**
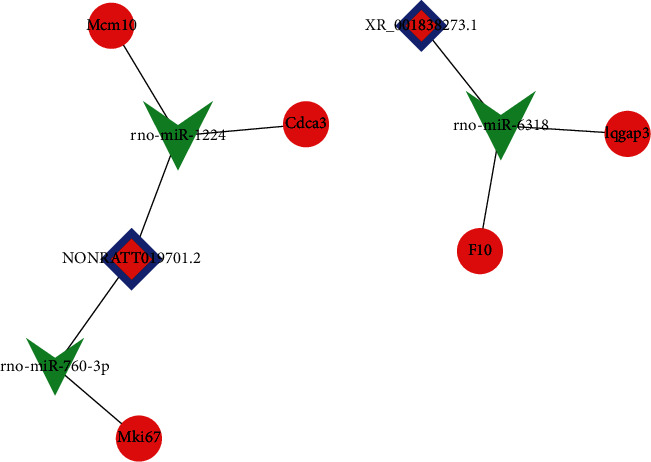
Key lncRNA-miRNA-mRNA interaction network. The red-blue rectangle represents lncRNAs, the red circle represents mRNAs, and the shape of green V represents miRNA.

We constructed a ceRNA network based on top 500 key lncRNA-mRNA coexpression pairs. This showed five pairs of ceRNA regulatory sequences, including two lncRNAs (NONRATT019701.2 and XR_001838273.1), three miRNAs (rno-miR-1224, rno-miR-760-3p, and rno-miR-6318), and five mRNAs, including *Cdca3*, *Mcm10*, *Mki67F10*, *Iqgap3*, and *F10* (Figure 6).

### 3.8. Verification of the Microarray Data by qPCR

**Figure 7:**
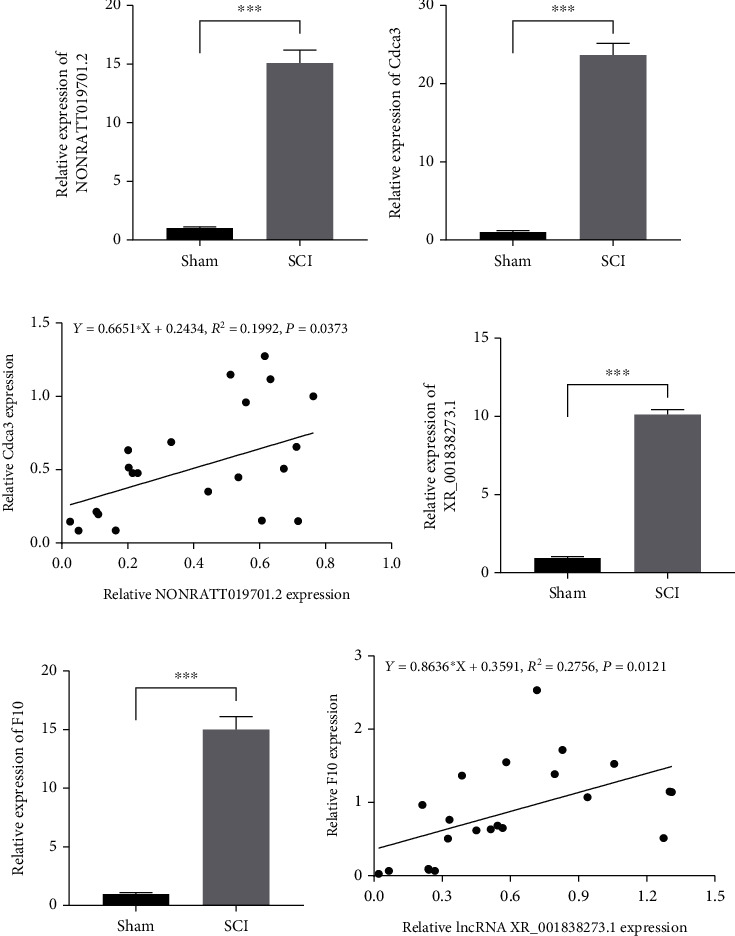
Validation of dysregulated RNAs by qRT-PCR. The key lncRNA (NONRATT019701.2 and XR_001838273.1) (a, d) and mRNA (Cdca3 and F10) (b, e) expressions in the SCI group (*n* = 3) and sham group (*n* = 3) were measured by qRT-PCR. Triplicate analyses were conducted on each sample. Correlation analysis between NONRATT019701.2 and Cdca3 and XR_001838273.1 and F10 expressions in the SCI rats (*n* = 22) (c, f). ^∗^*p* < 0.05, ^∗∗^*p* < 0.01, and ^∗∗∗^*p* < 0.001.

To verify the accuracy of the microarray data, the expression of several genes, including NONRATT019701.2, XR_001838273.1, *Cdca3*, and *F10*, in the SCI group (*n* = 3) compared with the sham group (*n* = 3) was analyzed by qPCR. The results showed that these genes were highly expressed (*p* < 0.05), consistent with the microarray analysis results, as shown in Figure 7. What is more, a positive correlation was found between the expression of the selected two pairs of lncRNA and mRNA in SCI rats (*n* = 22) by qRT-PCR.

## 4. Discussion

Researchers have recently focused on noncoding RNAs in SCI. It has been found that regulating the expression of noncoding RNA can reduce neuronal apoptosis, glial scar formation, and inflammation occurring during SCI and can improve SCI prognosis [18–21]. One study showed that overexpression of miR-182 improved lipopolysaccharide-induced apoptosis and inflammation BV2 cells; finding could block the IKK *β*/NF-*κ*B signaling pathway to inhibit cell apoptosis and the inflammatory response, thus improving the secondary injury caused by SCI [22]. Liu et al. found that exosomes secreted by hypoxic preconditioned mesenchymal stem cells shuttled miR-216a-5p to regulate microglial M2 polarization through the TLR4/NF-*κ*B/PI3K/AKT signaling axis [23] while Yoo et al. found that overexpression of miR-7 can reduce neuronal death and increase axonal growth, enhancing the recovery of motor function after SCI [18]. These findings suggest that noncoding RNAs have the potential for development as therapeutic targets for human SCI. It has been confirmed that lncRNAs are important regulators in the pathophysiology of many diseases, and researchers have also observed that SCI is associated with TFs, mRNAs, and signaling pathways that exert significant regulatory effects [19, 24, 25]. Therefore, this study is aimed at uncovering the broad characteristics of mRNA and lncRNA expressions and their interactions and coexpression networks and predicting the regulatory function of lncRNA in SCI.

This study examined lncRNAs and mRNAs in a rat model of SCI and identified 5465 lncRNAs and 8366 mRNAs that showed significantly different expression between the SCI and sham groups. It was found that “inflammatory response,” “positive regulation of NF-*κ*B transcription factor activity,” “positive regulation of interleukin-6 production,” and “Toll-like receptor signaling pathway”-related mRNAs were significantly upregulated. The KEGG analysis showed that the upregulated mRNAs were significantly associated with the “NF-*κ*B signaling pathway,” “Toll-like receptor signaling pathway,” “NOD-like receptor signaling pathway,” and “apoptosis.” It is known that the Toll-like receptor (TLR) activates inflammation in inflammatory reactions in various pathological conditions of the spinal cord [26]. Activation of TLR induces the production of IFN, cytokines, and chemokines and activates the NF-*κ*B signaling pathway [26]. For example, TLR4 mediates NF-*κ*B/IL-1*β* activation which can aggravate the inflammatory injury of the blood spinal cord barrier (BSCB) and nerve cells after spinal cord ischemia reperfusion [27]. Pattern recognition receptors (PPRs) recognize specific molecular patterns associated with foreign molecules, such as those expressed on the surfaces of pathogens, and thus play a key role in the innate immune response [28]. NOD-like receptor (NLR) is one of the main components of PPRs [28]. Activation of cytosolic NLR leads to the cleavage of caspase-1 and its conversion from an inactive state to an active enzyme [28, 29]. Activated caspase-1 promotes the production of inflammatory factors such as interleukin-18, which aggravate nerve damage [29]. Interestingly, both GO and KEGG analyses showed that the upregulated mRNAs were associated with immune inflammation. Similarly, the GSEA analysis also suggested that the principal changes after SCI involved pathways related to immune inflammation. It is thus apparent that the immune inflammatory response following SCI plays an important role in the development of the disease.

Many mechanisms are involved in regulating noncoding RNA expression, including chromatin regulation, epigenetic modifications, promoter activity regulation, and posttranscriptional modifications [30, 31]. lncRNAs are usually defined as a transcript with a length of more than 200 nucleotides and have no protein-coding function [30]. Most lncRNAs have not yet been clearly defined in their functions. Our aim was to further understand the function of lncRNAs by predicting their target genes based on “cis” and “trans” regulation. We found a total of 5465 DE lncRNAs in this study. In order to improve the accuracy of prediction, we conducted dimension reduction analysis through SVM-RFE and selected 40 key lncRNAs with high sensitivity and specificity. In the present study, we showed that the *Adgrg1* gene is a cis target gene of lncRNA NONRATT014027.2 and that the *NFATc4* gene is the cis target gene of lncRNA XR_359945.3. Chiou et al. found that knockout of *Adgrg1* in oligodendrocyte precursor cells significantly reduced the proliferation of these cells and reduced the number of myelinated axons [16]. Li et al. reported that overexpression of *NFATc4* in neuronal cells promotes neuronal apoptosis [17]. We speculate that lncRNA NONRATT014027.2 and lncRNA XR_359945.3 are both closely related to the proliferation and apoptosis of nerve cells.

The unannotated lncRNA XR_001840939.1 was predicted to cis regulate *Mark1* by interacting with nearby protein-coding genes. In addition, lncRNA XR_001840587.1 was predicted to cis regulate *Cblb.* Cell polarity cannot be established or maintained without MARK/PAR-1 family kinases [32]. It has been reported in the literature that DAPK DD binds to the spacer of MARK1 and MARK2, thereby relieving the autoinhibitory interaction between their N-terminal and C-terminal fragments [33]. The activation of MARK1/2 mediates the regulation of microtubule dynamics by DAPK while DAPK, in turn, promotes the regulation of polarized axon growth by MARK [33]. We speculate that lncRNA XR_001840939.1 may be involved in regulating microtubule dynamics. The *CBLB* gene encodes a multifunctional adapter protein that acts as a RING family E3 ubiquitin ligase that inhibits the activation of T cells and B cells [34, 35]. Additionally, when RNF125 and Cblb are present simultaneously, they target NLRP3 for sequential K63 and K48 polyubiquitination, which keeps inflammasomes in check by inhibiting their activity [36]. It is likely that lncRNA XR_001840587.1 is involved in immune inflammatory reactions in SCI.

As transmode RNAs, lncRNAs influence gene expression by binding to proteins, DNA, and other RNAs far from the main transcription sites [37]. In this study, lncRNA XR_359945.3 was predicted to transregulate *Zfyve27*, *KSR1*, *ELK4*, *Gfi1*, and *Apaf-1*. Gene-knockout studies have shown that *Gfi1* is essential for granulocyte development and plays a role in the production of macrophage-dependent cytokines, suggesting that the protein is responsible for defense against different pathogens [38]. Zheng et al. reported that ELK4 may reduce the PJA2-dependent occupation of KSR1 by translational activation of KDM5A to promote M2 macrophage polarization, leading to the development of gastric cancer [39]. ZFYVE27 was found to participate in neurite formation by the promotion of directional membrane transport [40]. In addition, overexpression of ZFYVE27 led to extensive axonal growth in PC12 cells and hippocampal neurons [40]. These findings suggest that ZFYVE27 plays an important role in neuronal development and differentiation. Previous studies have also reported that downregulation of *Apaf-1*, a key molecule in the intracellular apoptotic pathway, appears to be the preferred strategy to prevent nonmitotic cell apoptosis, which is achieved through endogenous inhibition, microRNAs, and epigenetic changes, amongst other mechanisms [41]. The expression of *Apaf-1* gradually decreases during brain development, enhancing the resistance of neurons to apoptosis [42]. Thus, lncRNA XR_359945.3 may be involved in the regulation of neuronal apoptosis, immune inflammation, and axonal regeneration.

This study also predicted regulatory transcription factors, and an lncRNA-TF-target gene network was constructed. The network showed several TFs that regulate the function of lncRNAs, such as Sp1, JUN, and SOX10. Sp1 functions in various physiological and pathological processes to bind DNA via its C2H2 zinc finger structure [43]. As shown by previous studies, Sp1 regulates the inflammatory response associated with many diseases [44, 45]. Qin et al. reported that preventing Sp1 activation can effectively inhibit OGD/R-induced microglial inflammatory activation [46]. JUN is a dimerization chaperone (Jun, Fos, and Atf families) and multimotif combiner (TRE, CRE, and some variants) and thus plays an important role in many biological processes, including proliferation, death, and regeneration [47]. In both rat CNS and mouse DRG neurons, JUN-ATF3 dimers promote neurite outgrowth [47]. A study on diabetes peripheral neuropathy by Jiao et al. found that miR-7a-5p regulation ameliorated mitochondrial dysfunction and reduced apoptosis via the VDAC1/JNK/c-JUN pathway [48]. The *SOX10* gene and SOX10 protein are responsible for the gliogenesis of glial cells in neural crest cells [49]. Furthermore, Xie et al. found that downregulation of Sp1 alleviated neuropathic pain-like behaviors after neutral alignment via the HDAC1/SOX10 axis [50]. Interestingly, we also found that Sp1, JUN, and SOX10 were involved in regulating XR_001837123.1 and Ets1. The lncRNAs associated with these three transcription factors may have similar functions. However, the specific function of these lncRNAs requires further investigation.

In the key lncRNA-ceRNA network, lncRNA NONRATT019701.2 acts as a sponge for rno-miR-1224 to regulate the target genes *Cdca3* or *Mcm10* or sponge for rno-miR-760-3p to regulate *Mki67*. lncRNA XR_001838273.1 acts as a sponge for rno-miR-6318 to regulate *Iqgap3* or *F10*. Numerous study literatures have reported that *Cdca3*, *Mcm10*, *Mki67*, *Iqgap3*, and *F10* are all related to tumor cell proliferation [51–56]. Knocking down the expression of these genes could reduce tumor cell proliferation and invasion. It has also been reported that the activity of PC12 cells in an oxygen-glucose deprivation/reoxygenation (OGD/R) model decreased after treatment with an miR-760-3p inhibitor [57]. Overexpression of miR-1224 inhibits hepatocyte proliferation and promotes apoptosis, according to Roy et al. [58]. A study by Wan and Xiao found that downregulating miR-1224 prevented neuronal apoptosis and mitochondrial damage caused by OGD/R [59]. We speculate that lncRNA NONRATT019701.2 and lncRNA XR_001838273.1 may have the potential to regulate the proliferation of microglia and glial cells. The increased expression of lncRNA NONRATT019701.2/XR_001838273.1 may aggravate the proliferation of microglia or glial cells and stimulate both the inflammatory reaction and scar formation.

## 5. Conclusion

In this study, we comprehensively analyzed both mRNAs and lncRNAs in SCI rats. Rats with SCI showed dysregulated mRNA and lncRNA expression profiles. Coexpression networks and SVM-RFE analyses demonstrated that several key lncRNAs regulated mRNA levels, and complex interactions between mRNA and lncRNA were also documented. Using bioinformatics approaches, we predicted the targets of DE lncRNAs and their potential functions. According to these findings, lncRNAs that are differentially expressed may play an important role in SCI. Further studies are needed to clarify how these mRNAs and lncRNAs contribute to SCI pathogenesis.

## Figures and Tables

**Table 1:** Top five upregulated and downregulated lncRNAs and mRNAs in the SCI group compared with the sham group.

lncRNA	*p* value	FC	Chromosome	Regulation
NONRATT006251.2	0.000042089	196.361	chr10	Up
XR_590951.1	0.000049424	123.494	chr2	Up
NONRATT021749.2	0.000739028	111.094	chr4	Up
NONRATT000346.2	0.000033721	96.565	chr1	Up
NONRATT006253.2	0.000031125	95.260	chr10	Up
XR_001838225.1	0.000009133	0.023237979	chr6	Down
NONRATT006738.2	0.000219361	0.022566731	chr11	Down
NONRATT014374.2	0.000043985	0.020947935	chr19	Down
NONRATT024533.2	0.000026402	0.016269598	chr6	Down
XR_001836692.1	0.000002296	0.014740753	chr2	Down
mRNA	*p* value	FC	Chromosome	Regulation
XM_006236630.3	0.000000002	875.0873735	chr4	Up
ENSRNOT00000009392	0.000139312	424.7759567	chr4	Up
XM_017592759.1	0.000009005	308.5014593	chr4	Up
ENSRNOT00000077186	0.000017596	248.0485524	chr1	Up
ENSRNOT00000077158	0.000003484	219.6519649	chr10	Up
ENSRNOT00000089734	0.000794572	0.020658751	chr7	Down
XM_006232396.2	0.000340082	0.017934131	chr2	Down
ENSRNOT00000034394	0.000121470	0.017696522	chr5	Down
ENSRNOT00000086508	0.000066847	0.017050394	chr13	Down
ENSRNOT000000881	0.000460103	0.016468971	chr3	Down

## Data Availability

The datasets presented in this study can be found in the GEO repository (accession number GSE218088).

## Supplementary Materials

Supplementary Materials Supplementary Table 1: the primer sequences used in this study for qRT-PCR. Supplementary Table 2: the 40 key lncRNAs screened using the SVM-RFE algorithm. Supplementary Figure 1: box plot showing the distribution of lncRNA expression values in 6 samples from the sham group (*n* = 3) and SCI group (*n* = 3). Supplementary Figure 2: GO and KEGG pathway functional enrichment analyses of the upregulated (A, B) and downregulated (C, D) DE mRNAs.

## References

[1] Ling Y. T., Alam M., Zheng Y.-P. (2020). Spinal cord injury: lessons about neuroplasticity from paired associative stimulation. *The Neuroscientist*.

[2] Pei J.-P., Fan L.-H., Nan K., Li J., Dang X.-Q., Wang K.-Z. (2017). HSYA alleviates secondary neuronal death through attenuating oxidative stress, inflammatory response, and neural apoptosis in SD rat spinal cord compression injury. *Journal of Neuroinflammation*.

[3] Liu H., Zhang J., Xu X. (2021). SARM1 promotes neuroinflammation and inhibits neural regeneration after spinal cord injury through NF-*κ*B signaling. *Theranostics*.

[4] Pelisch N., Rosas Almanza J., Stehlik K. E., Aperi B. V., Kroner A. (2020). CCL3 contributes to secondary damage after spinal cord injury. *Journal of Neuroinflammation*.

[5] Xu S., Wang J., Zhong J. (2021). CD73 alleviates GSDMD-mediated microglia pyroptosis in spinal cord injury through PI3K/AKT/Foxo1 signaling. *Clinical and Translational Medicine*.

[6] Jin W., Botchway B. O. A., Liu X. (2021). Curcumin can activate the Nrf 2/HO-1 signaling pathway and scavenge free radicals in spinal cord injury treatment. *Neurorehabilitation and Neural Repair*.

[7] Zhao H., Mei X., Yang D., Tu G. (2021). Resveratrol inhibits inflammation after spinal cord injury via SIRT-1/NF-*κ*B signaling pathway. *Neuroscience Letters*.

[8] Lin W., Zhou Q., Wang C.-Q. (2020). LncRNAs regulate metabolism in cancer. *International Journal of Biological Sciences*.

[9] Li Y., Yi M., Wang D., Zhang Q., Yang L., Yang C. (2020). LncRNA KCNQ1OT1 regulates endoplasmic reticulum stress to affect cerebral ischemia-reperfusion injury through targeting miR-30b/GRP78. *Inflammation*.

[10] Wang H., Zheng X., Jin J. (2020). LncRNA MALAT1 silencing protects against cerebral ischemia-reperfusion injury through miR-145 to regulate AQP4. *Journal of Biomedical Science*.

[11] Wucher V., Legeai F., Hedan B. (2017). FEELnc: a tool for long non-coding RNA annotation and its application to the dog transcriptome. *Nucleic Acids Research*.

[12] Alkan F., Wenzel A., Palasca O. (2017). RIsearch2: suffix array-based large-scale prediction of RNA-RNA interactions and siRNA off-targets. *Nucleic Acids Research*.

[13] Ren D., Chen W., Cao K., Wang Z., Zheng P. (2020). Expression profiles of long non-coding RNA and messenger RNA in human traumatic brain injury. *Molecular Therapy-Nucleic Acids*.

[14] Fornes O., Castro-Mondragon J. A., Khan A. (2020). JASPAR 2020: update of the open-access database of transcription factor binding profiles. *Nucleic Acids Research*.

[15] Yevshin I., Sharipov R., Kolmykov S., Kondrakhin Y., Kolpakov F. (2019). GTRD: a database on gene transcription regulation-2019 update. *Nucleic Acids Research*.

[16] Chiou B., Gao C., Giera S. (2021). Cell type-specific evaluation of ADGRG1/GPR56 function in developmental central nervous system myelination. *Glia*.

[17] Li L., Ke K., Tan X. (2013). Up-regulation of NFATc4 involves in neuronal apoptosis following intracerebral hemorrhage. *Cellular and Molecular Neurobiology*.

[18] Yoo M., Murphy A., Junn E. (2021). MicroRNA-7 promotes motor function recovery following spinal cord injury in mice. *Biochemical and Biophysical Research Communications*.

[19] Cui Y., Yin Y., Xiao Z. (2019). LncRNA Neat1 mediates miR-124-induced activation of Wnt/*β*-catenin signaling in spinal cord neural progenitor cells. *Stem Cell Research & Therapy*.

[20] Cao Y., Jiang C., Lin H., Chen Z. (2021). Silencing of long noncoding RNA growth arrest-specific 5 alleviates neuronal cell apoptosis and inflammatory responses through sponging microRNA-93 to repress PTEN expression in spinal cord injury. *Frontiers in Cellular Neuroscience*.

[21] Wang W., Liu R., Su Y., Li H., Xie W., Ning B. (2018). MicroRNA-21-5p mediates TGF-*β*-regulated fibrogenic activation of spinal fibroblasts and the formation of fibrotic scars after spinal cord injury. *International Journal of Biological Sciences*.

[22] Fei M., Li Z., Cao Y., Jiang C., Lin H., Chen Z. (2021). MicroRNA-182 improves spinal cord injury in mice by modulating apoptosis and the inflammatory response via IKK*β*/NF-*κ*B. *Laboratory Investigation*.

[23] Liu W., Rong Y., Wang J. (2020). Exosome-shuttled miR-216a-5p from hypoxic preconditioned mesenchymal stem cells repair traumatic spinal cord injury by shifting microglial M1/M2 polarization. *Journal of Neuroinflammation*.

[24] Xu S., Wang J., Jiang J. (2020). TLR4 promotes microglial pyroptosis via lncRNA-F630028O10Rik by activating PI3K/AKT pathway after spinal cord injury. *Cell Death & Disease*.

[25] Wang D., Chen F., Fang B. (2020). MiR-128-3p alleviates spinal cord ischemia/reperfusion injury associated neuroinflammation and cellular apoptosis via SP1 suppression in rat. *Frontiers in Neuroscience*.

[26] Newton K., Dixit V. M. (2012). Signaling in innate immunity and inflammation. *Cold Spring Harbor Perspectives in Biology*.

[27] Li X.-Q., Chen F.-S., Tan W.-F., Fang B., Zhang Z.-L., Ma H. (2017). Elevated microRNA-129-5p level ameliorates neuroinflammation and blood-spinal cord barrier damage after ischemia-reperfusion by inhibiting HMGB1 and the TLR3-cytokine pathway. *Journal of Neuroinflammation*.

[28] Mortezaee K., Khanlarkhani N., Beyer C., Zendedel A. (2018). Inflammasome: its role in traumatic brain and spinal cord injury. *Journal of Cellular Physiology*.

[29] Hung W.-L., Ho C.-T., Pan M.-H. (2020). Targeting the NLRP3 inflammasome in neuroinflammation: health promoting effects of dietary phytochemicals in neurological disorders. *Molecular Nutrition & Food Research*.

[30] Ransohoff J. D., Wei Y., Khavari P. A. (2018). The functions and unique features of long intergenic non-coding RNA. *Nature Reviews. Molecular Cell Biology*.

[31] Quinn J. J., Chang H. Y. (2016). Unique features of long non-coding RNA biogenesis and function. *Nature Reviews. Genetics*.

[32] Matenia D., Mandelkow E.-M. (2009). The tau of MARK: a polarized view of the cytoskeleton. *Trends in Biochemical Sciences*.

[33] Wu P. R., Tsai P. I., Chen G. C. (2011). DAPK activates MARK1/2 to regulate microtubule assembly, neuronal differentiation, and tau toxicity. *Cell Death and Differentiation*.

[34] Qiao G., Lei M., Li Z. (2007). Negative regulation of CD40-mediated B cell responses by E3 ubiquitin ligase Casitas-B-lineage lymphoma protein-B. *Journal of Immunology*.

[35] Schmitz M. L. (2009). Activation of T cells: releasing the brakes by proteolytic elimination of Cbl-b. *Science Signaling*.

[36] Tang J., Tu S., Lin G. (2020). Sequential ubiquitination of NLRP3 by RNF125 and Cbl-b limits inflammasome activation and endotoxemia. *The Journal of Experimental Medicine*.

[37] Wang D., Dai C., Zhang X. (2021). Identification and functional analysis of long non-coding RNAs in human pulmonary microvascular endothelial cells subjected to cyclic stretch. *Frontiers in Physiology*.

[38] Möröy T., Zeng H., Jin J., Schmid K. W., Carpinteiro A., Gulbins E. (2008). The zinc finger protein and transcriptional repressor Gfi1 as a regulator of the innate immune response. *Immunobiology*.

[39] Zheng L., Xu H., Di Y. (2021). ELK4 promotes the development of gastric cancer by inducing M2 polarization of macrophages through regulation of the KDM5A-PJA2-KSR1 axis. *Journal of Translational Medicine*.

[40] Shirane M., Nakayama K. I. (2006). Protrudin induces neurite formation by directional membrane trafficking. *Science*.

[41] Shakeri R., Kheirollahi A., Davoodi J. (2021). Contribution of Apaf-1 to the pathogenesis of cancer and neurodegenerative diseases. *Biochimie*.

[42] Fortin A., Cregan S. P., MacLaurin J. G. (2001). APAF1 is a key transcriptional target for p 53 in the regulation of neuronal cell death. *The Journal of Cell Biology*.

[43] Wang R., Yang Y., Wang H., He Y., Li C. (2020). MiR-29c protects against inflammation and apoptosis in Parkinson’s disease model in vivo and in vitro by targeting SP1. *Clinical and Experimental Pharmacology & Physiology*.

[44] Shin I.-S., Shin N.-R., Park J.-W. (2015). Melatonin attenuates neutrophil inflammation and mucus secretion in cigarette smoke-induced chronic obstructive pulmonary diseases via the suppression of Erk-Sp1 signaling. *Journal of Pineal Research*.

[45] Yang S., Yin J., Hou X. (2018). Inhibition of miR-135b by SP-1 promotes hypoxia-induced vascular endothelial cell injury via HIF-1*α*. *Experimental Cell Research*.

[46] Qin F., Zhao Y., Shang W. (2018). Sinomenine relieves oxygen and glucose deprivation-induced microglial activation via inhibition of the SP1/mi RNA-183-5p/I*κ*B-*α* signaling pathway. *Cellular and Molecular Biology*.

[47] Danzi M. C., Mehta S. T., Dulla K. (2018). The effect of Jun dimerization on neurite outgrowth and motif binding. *Molecular and Cellular Neurosciences*.

[48] Jiao Y., Zhang Y.-H., Wang C.-Y. (2023). MicroRNA‐7a‐5p ameliorates diabetic peripheral neuropathy by regulating VDAC1/JNK/c-JUN pathway. *Diabetic Medicine*.

[49] Bhattarai C., Poudel P. P., Ghosh A., Kalthur S. G. (2022). Comparative role of SOX10 gene in the gliogenesis of central, peripheral, and enteric nervous systems. *Differentiation*.

[50] Xie Y., Li Z., Xu H. (2022). Downregulation of Sp1 inhibits the expression of HDAC1/SOX10 to alleviate neuropathic pain-like behaviors after spinal nerve ligation in mice. *ACS Chemical Neuroscience*.

[51] Kang P., Han Z., Liao Z., Zhang H., Jia W., Tian Y. (2020). Knockdown of MCM10 gene impairs glioblastoma cell proliferation, migration and invasion and the implications for the regulation of tumorigenesis. *Journal of Molecular Neuroscience*.

[52] Zou R. C., Guo Z. T., Wei D. (2020). Downregulation of CDCA3 expression inhibits tumor formation in pancreatic cancer. *Neoplasma*.

[53] Hou Y.-Y., Cao W.-W., Li L. (2011). Micro RNA-519d targets MKi67 and suppresses cell growth in the hepatocellular carcinoma cell line QGY-7703. *Cancer Letters*.

[54] Song Y., Cao W., Zhu X. (2017). F10, a novel hydatidiform mole-associated gene, inhibits the paclitaxel sensitivity of A549 lung cancer cells by downregulating BAX and caspase-3. *Oncology Letters*.

[55] Matsuo J., Douchi D., Myint K. (2021). Iqgap3-Ras axis drives stem cell proliferation in the stomach corpus during homoeostasis and repair. *Gut*.

[56] Xu W., Xu B., Yao Y. (2016). Overexpression and biological function of IQGAP3 in human pancreatic cancer. *American Journal of Translational Research*.

[57] Zhang H., Deng J., Huang K., He Y., Cai Z., He Y. (2022). circNup188/miR-760-3p/Map3k8 axis regulates inflammation in cerebral ischemia. *Molecular and Cellular Probes*.

[58] Roy S., Bantel H., Wandrer F. (2017). mi R-1224 inhibits cell proliferation in acute liver failure by targeting the antiapoptotic gene Nfib. *Journal of Hepatology*.

[59] Wan J., Xiao T. (2022). MiR-1224 downregulation inhibits OGD/R-induced hippocampal neuron apoptosis through targeting Ku protein. *Metabolic Brain Disease*.

