# Supplementary material for: Expression Profiles of Long Noncoding RNAs and Messenger RNAs in a Rat Model of Spinal Cord Injury
Source: Comput Math Methods Med. 2023 Jan 19;2023:6033020. doi: 10.1155/2023/6033020 (PMC9879695; doi:10.1155/2023/6033020)

Supplementary Table 1| The primer sequences used in this study for qPCR.

| ID | Primer |
| --- | --- |
| LncRNA NONRATT019701.2 | forward: 5'-TCCCAACACTCTGTCAGCAACC-3'  reverse: 5'-CAGTGATGATGGAGCCAAACTC-3' |
| LncRNA XR_001838273.1 | forward: 5'-AAGGAATGCTCACCCCTCTGG-3'  reverse: 5'-GTGGGTCTCATTCAAGATGTCAGG-3' |
| Cdca3 | forward: 5'-TTGTCCAATCGGAAAGCAGAGA-3'  reverse: 5'-CAGCCATCCTTATTGGTTGACG-3' |
| F10 | forward: 5'-GTTACTTCCTGGGTAATGACGG-3'  reverse: 5'-TGAGGTCTTCAGGGTCAGGTTC-3' |
| GAPDH | forward: 5'-CTGGAGAAACCTGCCAAGTATG-3'  reverse: 5'-GGTGGAAGAATGGGAGTTGCT-3' |

Supplementary Table 2| The 40 key lncRNAs screened using the SVM- RFE algorithm.

| **No.** | **lncRNA** | **No.** | **lncRNA** |
| --- | --- | --- | --- |
| 1 | NONRATG009350.2 | 21 | NONRATG006341.2 |
| 2 | XR_359945.3 | 22 | NONRATG021098.2 |
| 3 | XR_001840872.1 | 23 | NONRATG022948.2 |
| 4 | NONRATG021777.2 | 24 | NONRATG014393.2 |
| 5 | NONRATG017244.2 | 25 | XR_001840587.1 |
| 6 | NONRATG000561.2 | 26 | NONRATG023453.2 |
| 7 | NONRATG023345.2 | 27 | NONRATG017743.2 |
| 8 | XR_001837123.1 | 28 | XR_352994.3 |
| 9 | NONRATG019728.2 | 29 | NONRATG016082.2 |
| 10 | NONRATG000285.2 | 30 | NONRATG011453.2 |
| 11 | XR_594211.2 | 31 | XR_001838273.1 |
| 12 | NONRATG009771.2 | 32 | NONRATG013745.2 |
| 13 | XR_001841055.1 | 33 | NONRATG015696.2 |
| 14 | ENSRNOG00000051412 | 34 | XR_001836613.1 |
| 15 | NONRATG002650.2 | 35 | ENSRNOG00000055234 |
| 16 | NONRATG005027.2 | 36 | NONRATG022639.2 |
| 17 | NONRATG015249.2 | 37 | NONRATG014456.2 |
| 18 | NONRATG003940.2 | 38 | NONRATG019006.2 |
| 19 | NONRATG021919.2 | 39 | ENSRNOG00000055201 |
| 20 | NONRATG017052.2 | 40 | XR_001840939.1 |

Supplementary Figure 1| Box plot showing the distribution of lncRNA expression values in 6 samples from the sham group (n=3) and SCI group (n=3).


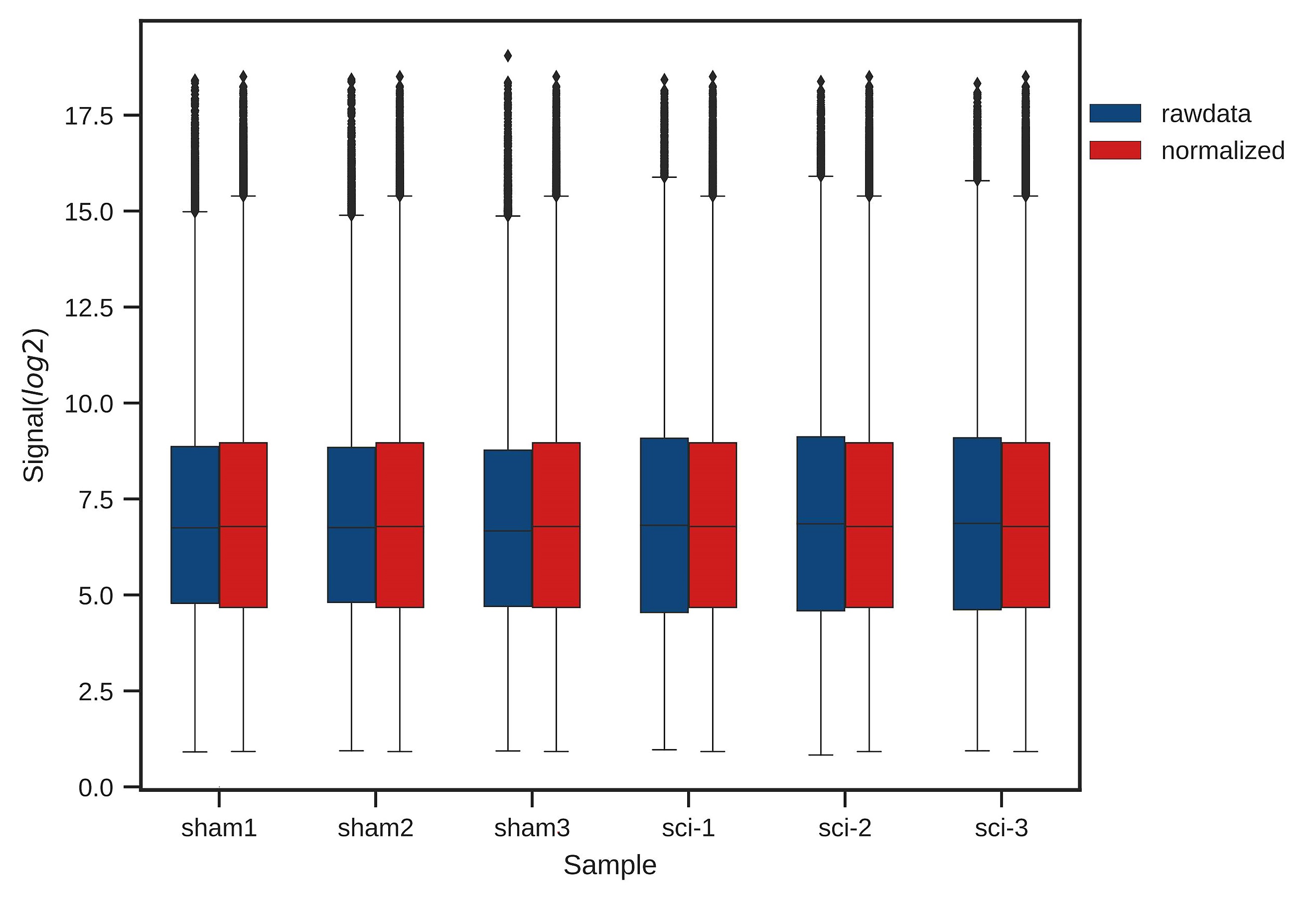


Supplementary Figure 2| GO and KEGG pathway functional enrichment analysis of the upregulated and downregulated DE mRNAs. Top 10 enriched GO terms in the molecular functions, biological processes, and cellular component categories for the upregulated and downregulated DE mRNAs (A and C) and top 30 enriched KEGG pathways for upregulated and downregulated DE mRNAs (B and D).


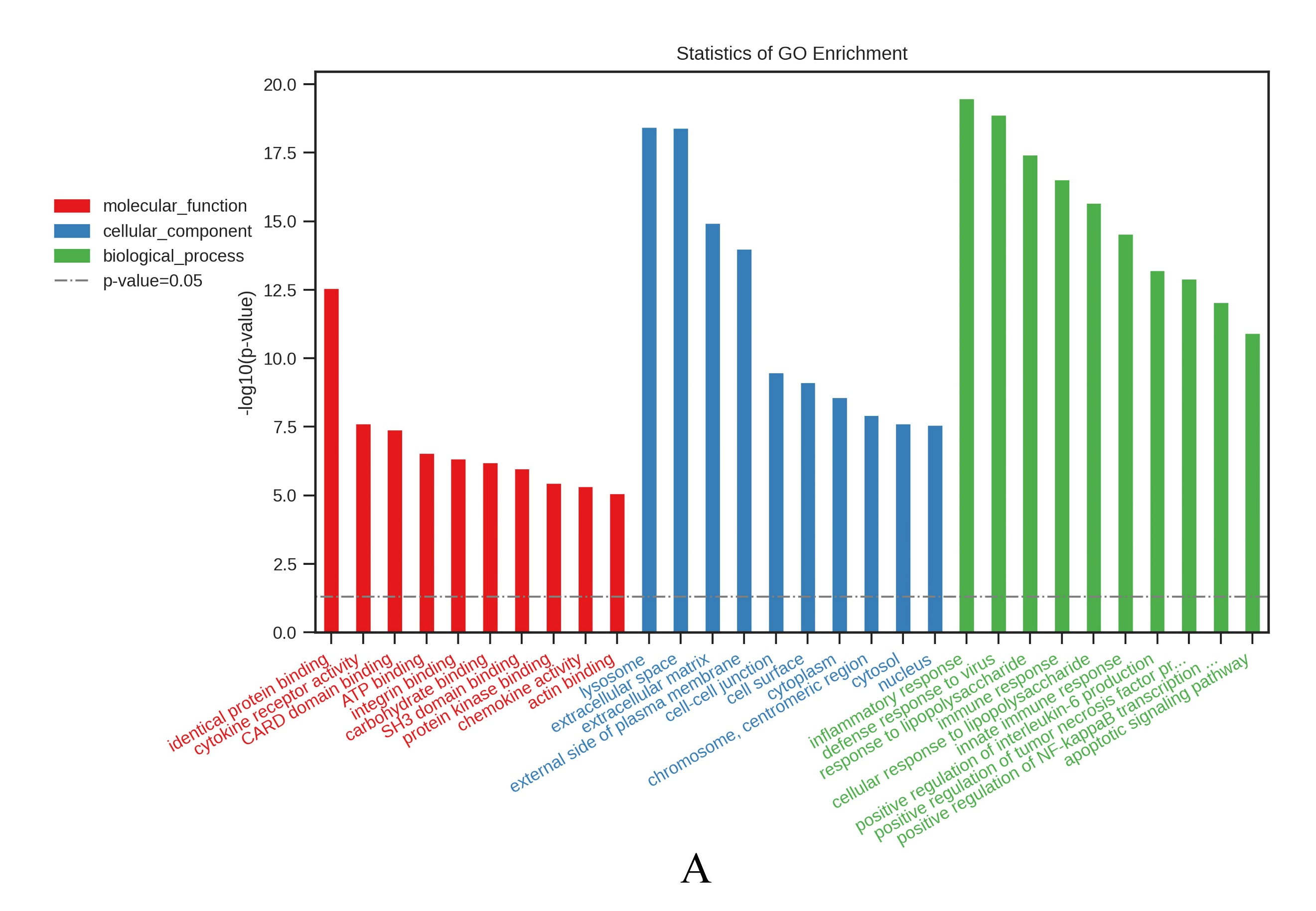


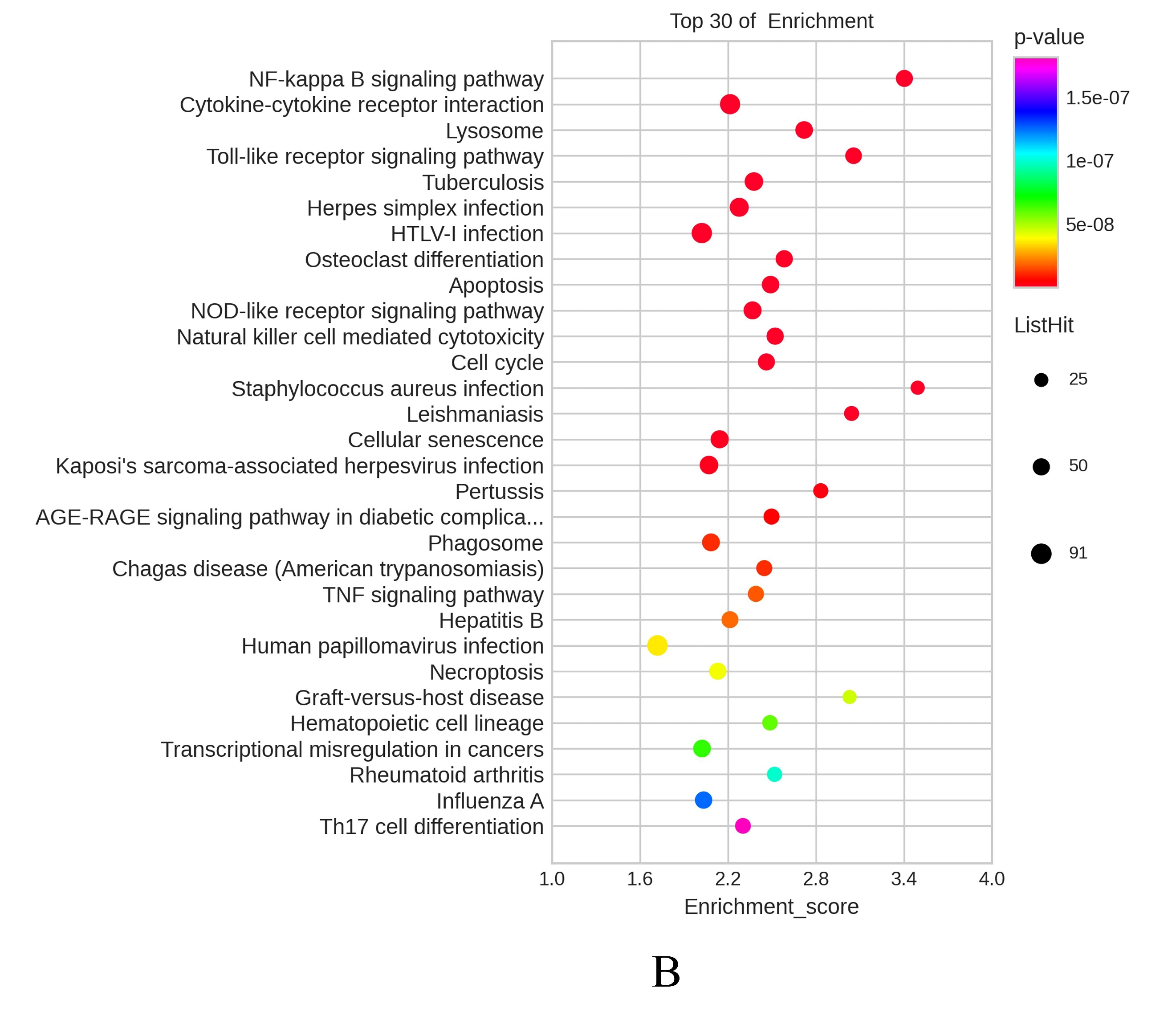


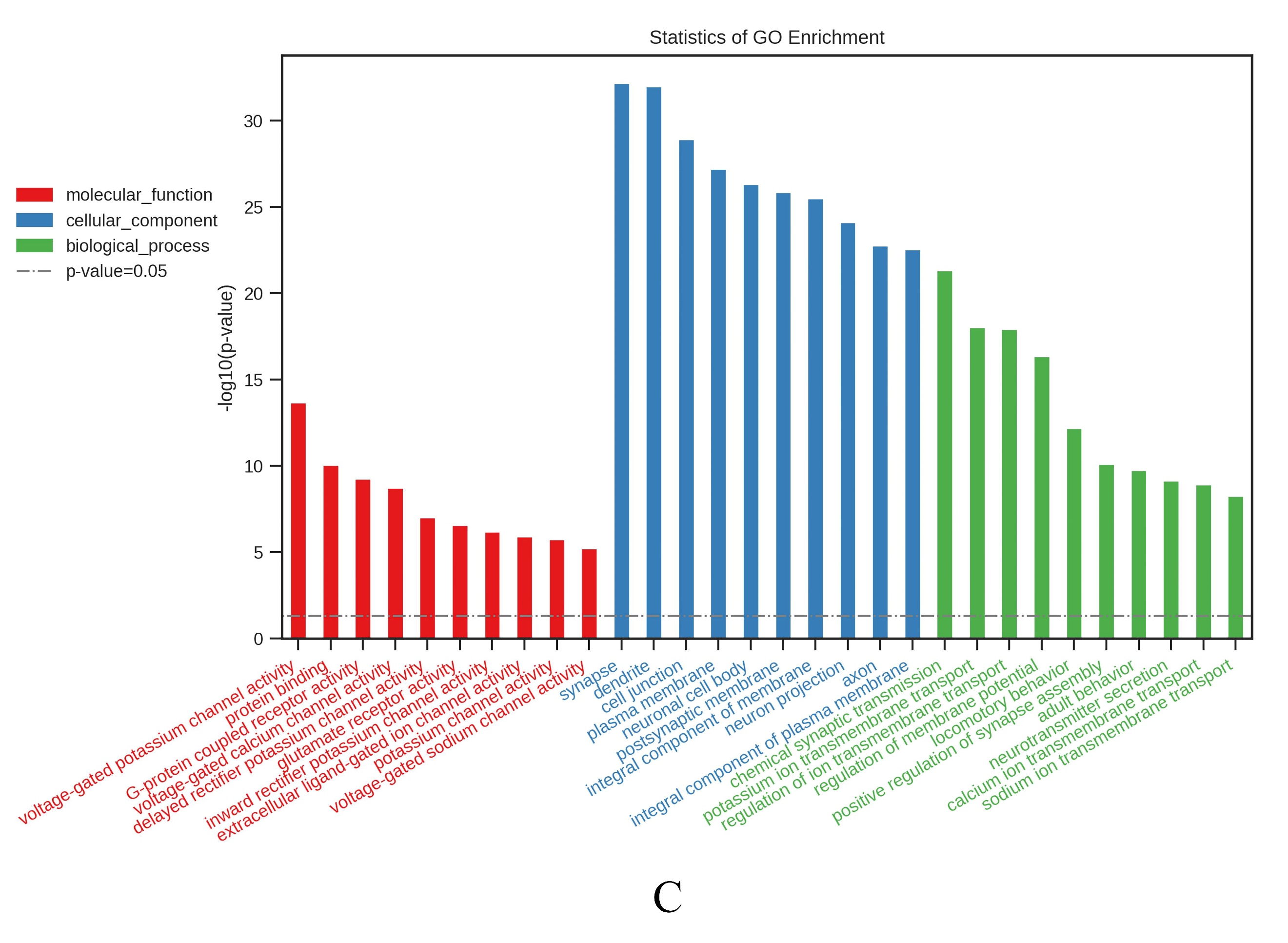


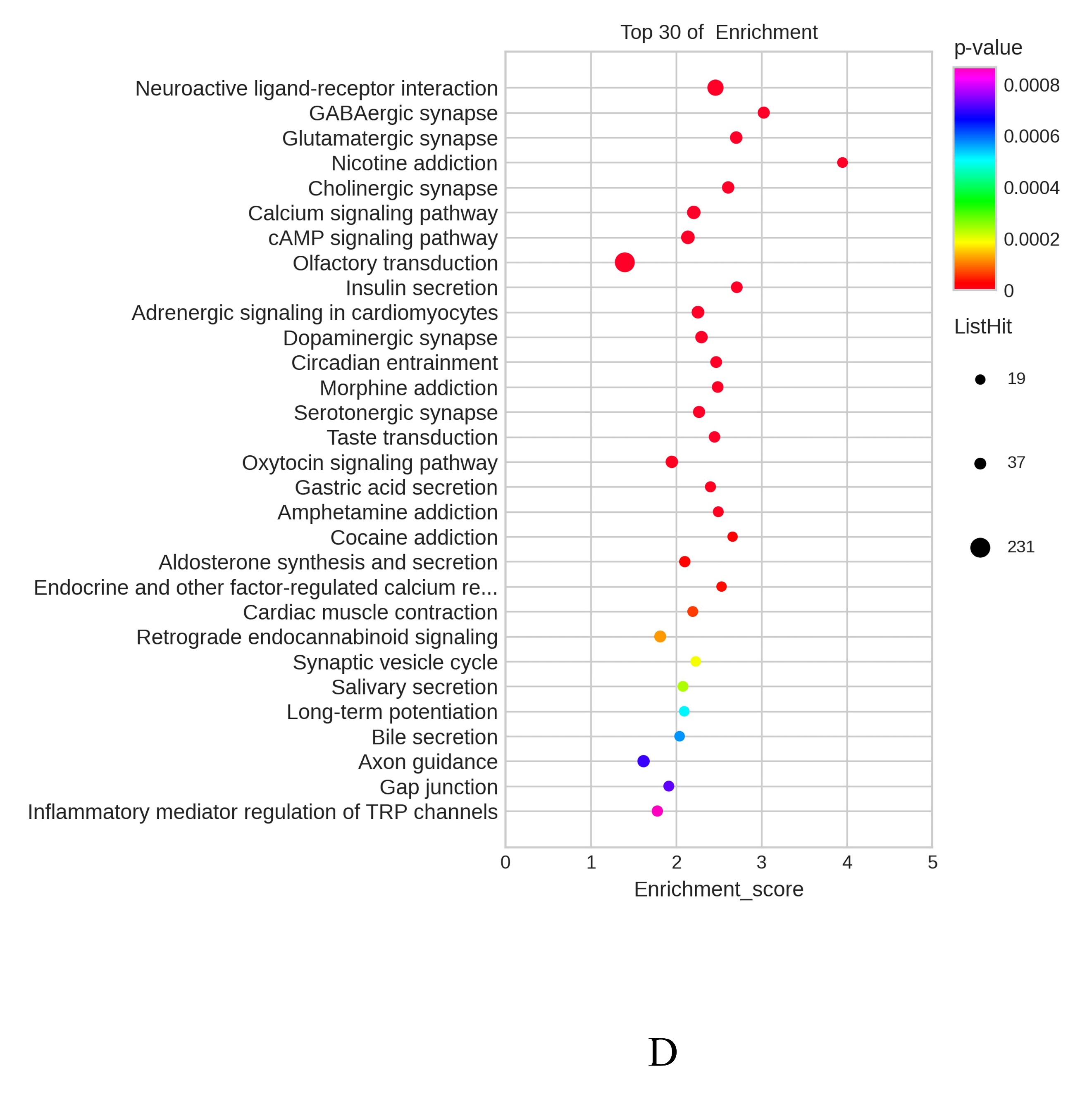

Supplement: Supplementary Materials — Supplementary Table 1: the primer sequences used in this study for qRT-PCR. Supplementary Table 2: the 40 key lncRNAs screened using the SVM-RFE algorithm. Supplementary Figure 1: box plot showing the distribution of lncRNA expression values in 6 samples from the sham group (n = 3) and SCI group (n = 3). Supplementary Figure 2: GO and KEGG pathway functional enrichment analyses of the upregulated (A, B) and downregulated (C, D) DE mRNAs. [file 6033020.f1.docx]
